# Evaluation of long-term outcomes associated with extended heavy-silicone oil use for the treatment of inferior retinal detachment

**DOI:** 10.1038/s41598-022-15896-y

**Published:** 2022-07-08

**Authors:** Fatih Horozoglu, Hidayet Sener, Osman Ahmet Polat, Ozkan Sever, Busra Potoglu, Erkan Celik, Elif Betul Turkoglu, Cem Evereklioglu

**Affiliations:** 1 Department of Ophthalmology, Erciyes University School of Medicine, Kayseri, Turkey; 2 Department of Ophthalmology, Cukurca State Hospital, Hakkari, Turkey; 3 Department of Ophthalmology, Silivri Kolan Hospital, Istanbul, Turkey; 4 Department of Ophthalmology, Namık Kemal University School of Medicine, Tekirdag, Turkey; 5 Department of Ophthalmology, Sakarya University School of Medicine, Sakarya, Turkey; 6 Department of Ophthalmology, Akdeniz University School of Medicine, Antalya, Turkey

**Keywords:** Outcomes research, Risk factors

## Abstract

To evaluate macular status with optical coherence tomography (OCT) in eyes that underwent pars plana vitrectomy (PPV) and heavy-silicone oil (HSO) endotamponade for the treatment of rhegmatogenous retinal detachment (RRD) with inferior breaks. Twenty eyes of 20 patients who have RRD with inferior breaks included in the study. Oxane HD was used as an intraocular tamponade for all surgeries. Postoperatively, anatomic reattachment, macular status using OCT imaging, and any long-term complications were evaluated. The mean age was 60.4 ± 11.2 years (range, 37–83). The duration of HSO endotamponade was 15.3 ± 11.0 months (range, 6–48) with some postoperative complications such as HSO emulsification, intraocular pressure elevation, and epiretinal membrane (ERM) formation. Mean follow-up time was 19.5 ± 10.5 months (range, 10–59) after HSO removal or ERM surgery. Primary reattachment was achieved in 90% of eyes and the success rate was 100% with further interventions. Ellipsoid zone (EZ) was continuous in 13 of 20 eyes in which OCT imaging performed as well as the fellow eye. PPV and heavy-silicone oil injection for the treatment of eyes with RRD from inferior break(s) have a good long-term EZ continuity. ERM formation and its removal do not affect EZ.

## Introduction

Management of rhegmatogenous retinal detachment (RRD) with pars plana vitrectomy (PPV) has evolved in the past decade with the invention of smaller transconjunctival incisions and increased vitreous cut rates^1^. Despite these advances, RRD with inferior breaks especially with proliferative vitreoretinopathy (PVR) is still challenging because of difficulty in tamponading the inferior retina. Although intravitreal gas may be used as a tamponade for inferior breaks, it is difficult to maintain postoperative positioning. Heavy-silicone oil (HSO) tamponade is another choice in treating inferior breaks without postoperative positioning as it has a specific gravity heavier than water. Oxane HD is a HSO that is reported to be effective in treating inferior RRD. Many unfavorable postoperative complications have been reported to be associated with Oxane HD, such as cataract, emulsification of HSO, ocular hypertension, retinal detachment and PVR, severe intraocular inflammation or uveitis, retro-oil epiretinal membranes (ERM), intraretinal or subretinal fibrosis^2^. Additionally, unexplained visual loss following the removal of HSO has been reported^3^. Using optical coherence tomography (OCT), we aimed to evaluate the long-term macular status of patients who underwent with PPV and extended HSO tamponade for treatment of RRD with inferior breaks.

## Methods

All procedures performed in human participants were in accordance with the ethical standards of the Namık Kemal University Local Ethics Committee and with the 1964 Helsinki declaration and its later amendments or comparable ethical standards. Experimental/study protocol was approved by Namık Kemal University Local Ethics Committee (No: 2017/81/08/05). Informed consent was obtained from all individual participants included in the study.

Twenty eyes of 20 patients who were treated with PPV and HSO (Oxane-HD, Bausch-Lomb, USA) endotamponade for RRD with inferior break(s) were evaluated from August 2011 to December 2020. We evaluated all patients who had OCT **(**Cirrus HD-OCT, Carl Zeiss Ophthalmic System Inc, USA**)** after the removal of HSO. Informed consent was signed by all patients before the operation.

Patients with RRD and inferior break(s) and a PVR level of B or more as graded using the Retina Society Classification^4^ were included in the study. Exclusion criteria were as follows: the eyes with (1) uveitis or any inflammatory disease; (2) retinal vascular disease; (3) penetrating trauma; (4) previous PPV with silicone oil injection.

Best-corrected visual acuity on Snellen chart, slit-lamp examinations of the anterior and posterior segments with both non-contact and contact lenses, and the measurement of intraocular pressure (IOP) were performed. All patients had OCT evaluation at the last visit by an experienced technician. High Definition (HD) 5 Line Raster spaced at 0.25 mm was centered on the fovea with 6-mm parallel lines, 1024 A-scans/B-scans and an average of 4 B-scans per image. Three consecutive measurements were taken at the fovea. The average of three measurements was used for analysis. Any error in segmentation was corrected by a retina specialist (F.H.).

All surgeries were performed under subtenon’s anesthesia by the same experienced surgeon (F.H.). All patients had 23/25-gauge transconjunctival sutureless vitrectomy. The standard surgical procedure was core vitrectomy, removing epiretinal or subretinal membranes if there was proliferation, retinotomy if needed, perfluorocarbon liquid and/or fluid-air exchange and injection of HSO. Phakic eyes had combined surgery with phacoemulsification and intraocular lens implantation and PPV. HSO was removed using an 18-gauge cannula with another PPV operation in all eyes under subtenon’s anesthesia. ERM surgeries were performed using Brilliant blue (Meran ILM dye, Turkey) and the internal limiting membrane (ILM) was removed from all eyes.

Statistical analysis was performed using SPSS version 22 (IBM, USA). Shapiro–Wilk normality test and descriptive statistics were performed on the data set. Since the data were normally distributed, one-sample t-test, dependent samples t-test, independent samples t-test, regression analysis and Pearson correlation analysis were performed as indicated. Since the time of presence of silicone did not fit the normal distribution, statistical normalization was performed with 1/data transformation and regression analysis was performed.

## Results

Twenty eyes (13 right, 7 left) of 20 patients (19 men, 1 woman) with RRD from inferior break(s) were included in this study. The average age was 60.4 ± 11.2 years (range, 37–83 years). The macula was detached in 8 eyes and was attached in 12 eyes. All 20 eyes had RRD with inferior breaks. None of the patients had high myopia. Eight eyes were phakic and 12 eyes were pseudophakic. The eight phakic eyes had combined phacovitrectomy because a phacovitrectomy has been reported to yield better anatomical results in phakic eyes with RRD^5^, which required no grading of the lens status per-surgery.

The average follow-up time after silicone oil removal or ERM surgery was 19.5 ± 10.5 months (range, 10–59). The duration of HSO endotamponade was 15.3 ± 11.0 months (range, 6–48). HSO cannot be removed early in these patients for various reasons including poorly cooperation, missing visits, and funding problems which even makes routine examination and follow up a challenge.

**Figure 1:**
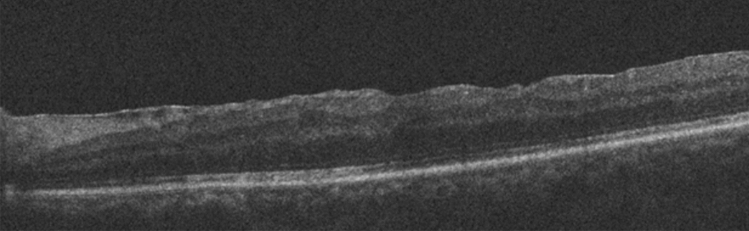
Postoperative OCT image of a case with off-macular detachment.

**Figure 2:**
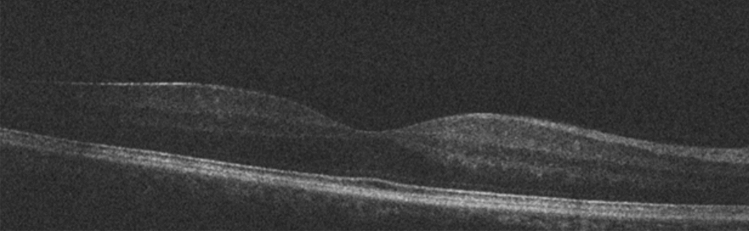
Fellow eye OCT image of a case with off-macular detachment.

Retinal reattachment was achieved in 18 eyes (90%) with a first operation. Recurrent detachment with PVR occurred after HSO removal in 2 eyes and retinal reattachment was achieved in these two eyes after second operations. ILM was removed in both 2 eyes with recurrence. The macular OCT data were obtained and evaluated postoperatively. The average central foveal thickness (CFT) was 191.7 ± 57.5 µm (range, 100–274 µm). No significant difference (*p* = 0.065) was found in the comparison between the average CFT in our study and the reference average CFT^6^ in the literature. The postoperative OCT image of a case with off-macular detachment is presented in Fig. 1 and the fellow eye image in Fig. 2. Ellipsoid Zone (EZ) was continuous in eyes that OCT evaluation was performed (13 of 20 eyes). Fellow eye of the patients had also continuous EZ.

The formation of ERM occurred in 10 eyes (50%). In 5 eyes with ERM, both HSO removal and ERM surgery were performed in the same session. ERM surgery was performed in 3 eyes with ERM in the first 3 months after HSO removal and in 1 eye with ERM between the 3rd and 6th months after HSO removal. ERM surgery was not performed in one eye with ERM. In one eye, F4H5 Washout (Fluoron Gmbh, Germany) was used to remove emulsified silicone oil droplets after ERM surgery. When the CFT was evaluated in patients who had and had not ERM surgery, it was observed that the average CFTs were 162.8 ± 50.5 µm and 225.5 ± 48.4 µm, respectively. When the correlation between ERM surgery and CFT was evaluated, a statistically significant negative moderate correlation was found (*p* = 0.044, r =  − 0.565). According to simple linear regression analysis, performing ERM surgery in foveal thinning explained 31.9% of the variance (R^2^ = 0.319, β =  − 62.6, 95% CI [− 123.4, − 1.8], *p* = 0.044). Eyes with emulsified silicone and eyes with non-emulsified silicone were found to be insignificant when high IOP and ERM were compared (*p* = 0.463, *p* = 0.179, respectively).

When preoperative (1.26 ± 0.83) and final (0.73 ± 0.51) visual acuities were compared, it was found that there was a statistically significant increase in final visual acuity (*p* = 0.009). When the preoperative and postoperative visual acuities of patients with macular detachment were compared, a statistically significant improvement was observed in visual acuity (*p* = 0.018), while there was no statistically significant increase in visual acuity in the eyes that had ERM surgery (*p* = 0.263).

Increased IOP (> 21 mmHg) was observed in 11 eyes, which was controlled by medical treatment. Moderate aqueous inflammation was observed in one eye but fibrinous exudation and synechia were not observed. Finally, emulsification of HSO was observed in 13 eyes. No other postoperative complications were observed in the patients. The complication rates of the case series are presented in Table 1. Clinical characteristics of the patients are presented in Table 2.

**Table 1:** The frequency of complications in patients with long-term heavy silicone oil implantation after primary PPV operation.

Complication	Rate
Raised IOP (> 21 mmHg**)**	11 (55%)
Emulsification	13 (65%)
Epiretinal Membrane	10 (50%)
Inflammation	1 (5%)
Recurrence of detachment	2 (10%)

**Table 2:** Clinical characteristics of the patients.

Patient	Age/gender	Lens status	PVR grade	Time of removal silicone oil (months)	Follow up time (months)	Preop VA (logMAR)	Final VA (logMAR)	Preop IOP (mmHG)	Postop IOP (mmHG)	Preop macula	Redetachment	Complication	Foveal thickness (µm)
1	61/M	Phakic	C	8	16	1.3	1.0	< 21	> 21	Off	No	ERM, Emulsification	222
2	83/M	Pseudophakic	C	24	16	2.0	1.03	< 21	> 21	Off	No	Emulsification	140
3	54/M	Phakic	B	9	13	1.8	0.4	< 21	< 21	Off	No	Emulsification	268
4	37/M	Pseudophakic	B	6	18	2.3	0.8	< 21	> 21	Off	No	Emulsification	NA
5	57/M	Pseudophakic	B	16	24	2.3	0.7	< 21	> 21	Off	No	Emulsification	NA
6	60/M	Pseudophakic	C	8	18	0.3	0.4	< 21	< 21	On	No	None	274
7	65/M	Phakic	C	12	59	0.8	0.1	< 21	< 21	On	No	Emulsification	236
8	73/M	Pseudophakic	C	16	18	0.1	0.18	< 21	< 21	On	No	Emulsification	213
9	55/F	Pseudophakic	B	48	30	0.3	0.1	< 21	> 21	On	No	None	NA
10	53/M	Pseudophakic	B	8	24	1.0	0.7	< 21	> 21	On	No	Emulsification	NA
11	53/M	Pseudophakic	B	8	24	1.0	0.7	< 21	> 21	On	No	Emulsification	NA
12	74/M	Pseudophakic	B	16	10	2.3	2.3	< 21	< 21	Off	Yes	ERM, Emulsification	NA
13	51/M	Phakic	C	16	15	1.7	0.7	< 21	< 21	Off	No	ERM	100
14	71/M	Phakic	C	16	12	2.6	0.7	< 21	< 21	Off	Yes	ERM	191
15	62/M	Pseudophakic	B	7	16	2.3	0.5	< 21	< 21	On	No	ERM, Emulsification	119
16	44/M	Phakic	C	8	15	1.3	0.8	< 21	> 21	On	No	ERM	125
17	67/M	Pseudophakic	B	28	21	0.18	0.4	< 21	> 21	On	No	ERM, Emulsification	221
18	70/M	Phakic	B	9	12	0.7	0.4	< 21	< 21	On	No	ERM, Inflammation	159
19	68/M	Pseudophakic	B	36	16	0.4	1.3	< 21	> 21	On	No	ERM, Emulsification	NA
20	50/M	Phakic	B	8	14	0.7	1.3	< 21	> 21	On	No	ERM	225

PVR: Proliferative vitreoretinopathy, ERM: epiretinal membrane.

According to the binary logistic regression analysis performed, no correlation was found between the time of presence of silicone in the eye and its emulsification (*p* = 0.396). According to the simple linear regression analysis performed, no correlation was found between the time of presence of silicone in the eye and the thickness of the center of the fovea (*p* = 0.715).

## Discussion

The use of internal tamponade is a challenging situation in eyes with inferior RRD. Prone positioning should be given after the use of conventional silicone oil as well as intraocular gas to tamponade the inferior retina in order to avoid an unsupported retinal area by upright positioning. Due to various reasons, however, tamponade of the lower retina may not be effective and sufficient with standard silicone oil. Many studies have found that HSO is effective and safe in the treatment of inferior retinal detachment^7,8^. However, in many studies, HSO was left in the eye as an endotamponade for 3–6 months^7,9,10^. There are studies evaluating long-term effects, but cases with additional interventions were evaluated in these reports^11,12^.

In our study, we used HSO as the primary endotamponade in cases of RRD with inferior breaks of poorly cooperative patients and evaluated the long-term results of HSO. Extended HSO usage did not have a negative effect on postoperative visual acuity. In turn, it was improved in 15 eyes (75%) and better or equal to 20/200 in 16 eyes (80%). There was also improvement in visual outcomes in patients with macular detachment. Our visual results are comparable and acceptable to other studies^11,12^. In addition, long-term HSO usage did not affect CFT. In their study using OCT, Hostovsky et al.^13^, observed that HSO, which was remained in the eye for up to 6 months, caused temporary and reversible retinal thinning, attributed to the mechanical effect. It is reported that short-term use can prevent long-term changes in the fovea^13^. In addition, it has been reported that silicone oil increases macular thickness in diabetic patients, while it causes retinal thinning in non-diabetic patients, and it has been reported that these structural changes may regress after silicone removal^14^. Disorganization of the outer plexiform layer, photoreceptors and RPE has been reported due to both mechanical stress and toxic effects of silicone oils^15–17^. However, we observed that EZ continuity was well preserved in the long term.

In the literature, it is observed that the most common complication developing after HSO is cataracts^2,18^. However, in our series, 40% of the cases were phakic and cataract surgery was performed during primary PPV. The most common complication in our series was the emulsification of HSO in 65% of cases, the rate of which was higher than those previously reported^2,10,11,19–21^. Although there was no relationship between the presence time of silicone in the eye and emulsification in the regression analysis, it is known that the main factor affecting the tendency to emulsify is the time elapsed until the removal of these tamponades^22^. The higher rate of emulsification compared to the literature may be the result of long-term presence of HSO in the eye. Due to emulsified silicone oil droplets in one patient, vitreous cavity was cleaned using a perfluorobutylpentane (F4H5) solvent-assisted silicon oil removal technique^23^.

The second most common complication is elevated IOP. In our series, the rate of elevated IOP was higher than in the literature^2,11,12,18^. Although we have found that eyes between emulsified and non-emulsified silicone oil were similar in terms of IOP elevation and ERM formation, emulsified silicone oil has been reported to give predisposition to such complications^21,24^. Another reason could be overfilling. The elevated IOP in our series was controlled with medical therapy and none of eyes required surgery. ERM development was higher compared to the literature^11,12,18^. An inflammatory reaction due to the use of HSO, emulsified silicone, or accumulation of preretinal liquid with high inflammatory agents trapped between retina and HSO may be the precursor of this condition^25^. In addition, it is known that with the use of HSO, the preretinal liquid that is concentrated in inflammatory agents is pushed upwards in the horizontal axis, the ERM is concentrated above and the posterior pole is protected^26^. Like previous studies, therefore, inflammation and long term presence of HSO may be the reason of this higher ERM rates.

The advantages of this study are; it reflects the experience of a single surgeon with a single HSO endotamponade as a primary surgery. The limitations of the study are its retrospective design and the small number of patients. However, our study is the first to give results on long-term HSO usage, as well as EZ continuity, according to our knowledge.

In conclusion, the present report revealed that long-term use of HSO for the treatment of RRDin eyes with inferior breaks resulted in good anatomical reattachment of the retina and satisfactory functional results. In addition, PPV and HSO injection in such cases have good EZ continuity and foveal thickness in the long-term manner. However, extended use of HSO increases the rate of emulsification, IOP elevation and ERM formation. Despite the high complication rate, it is possible to preserve HSO in the eyes for a long time in a small group of uncooperative patients, which prevent multiple operations and the risk of hypotonia^27^. Prospective studies with larger series are needed to further characterize the efficacy and safety of extended use of HSO in RRD with inferior break(s).

## Data Availability

The datasets generated during and/or analysed during the current study are available from the corresponding author on reasonable request.
